# Biochemical and Molecular Characterization of *Pichia pastoris* Cells Expressing Multiple TMOF Genes (*tmf*A) for Mosquito Larval Control

**DOI:** 10.3389/fphys.2020.00527

**Published:** 2020-05-26

**Authors:** Dov Borovsky, Sabine Nauwelaers, Robert Shatters

**Affiliations:** ^1^Department of Biochemistry and Molecular Genetics, University of Colorado Anschutz School of Medicine, Aurora, CO, United States; ^2^Zoological Institute, KU Leuven, Leuven, Belgium; ^3^USDA ARS, Subtropical Horticultural Laboratory, Fort Pierce, FL, United States

**Keywords:** genetic engineering, *Pichia pastoris*, low and high volume fermentations, TMOF, larval control

## Abstract

Trypsin modulating oostatic factor (TMOF), a decapeptide hormone synthesized by female mosquito ovaries, ganglia and the central nervous system of *Aedes aegypti*, terminates trypsin biosynthesis in larvae, and blood-fed female mosquitoes. Earlier, TMOF was cloned and expressed as a single copy in *Chlorella dessicata* and in *Saccharomyces cerevisiae* cells as a potential larvicide. Here we report the use of a methylotrophic yeast cells, *Pichia pastoris*, that efficiently express multi copies of heterologous proteins, that are readily ingested by mosquito larvae. *P. pastoris* was engineered using pPICZB (Invitrogen, CA, United States), and 2 genes: *gfp-tmf*A and *tmf*A inserted between *Kpn*I and *Xba*I in the multiple cloning site. The plasmid carries a strong AOXI promoter and *P. pastoris* KM71 and KM71H cells were transformed by homologous recombination. The synthesis of GFP-TMOF was followed using UV and clones were analyzed using southern and Northern blot analyses. Cloning *tmf*A into KM71H and selection on high Zeocin concentration (2.0 mg/mL) identified a clone that carried 10 copies of *tmfA.* A comparison between a single and high copy (10 genes) insertions using Northern blot analyses showed that a *tmfA* transcript was highly expressed even after 120 h. SDS-PAGE analysis of KM71 cells transformed with *gfp-tmf*A identified a protein band that ran at the expected *M*_*r*_ of 31 kDa. Enzyme Linked Immunoadsorbant Assay (ELISA) analysis of the recombinant cells showed that 1.65 × 10^8^ and 8.27 × 10^7^ cells produce 229 and 114 μM of TMOF, respectively, and caused 100% larval mortality when fed to groups of 5 larvae in 25 mL water. These results indicate that the recombinant *P. pastoris* cells could be used in the future in the marsh to control mosquito populations.

## Introduction

Environmental, resistance and human health concerns for using chemical pesticides have been a major reason in searching for new biorational insecticides to battle pests such as mosquitoes, transmitters of several detrimental human diseases including malaria, dengue, yellow fever, encephalitis (Spielman and D’Antonio, 2001), and Zika virus. These diseases cause health problem worldwide and Malaria alone causing death to more than one million people in Africa including 300–500 million clinical cases annually (World Health Organization [WHO], 1998). Traditional controls using chemical insecticides to control mosquitoes and agricultural pests often cause environmental and human health problems, as well as, the development of resistance. Thus, new approaches are urgently needed to overcome these challenges to health.

One approach is to utilize insect-specific peptide hormones to specifically control insects. These peptide hormones control multiple functions in insects; digestion, reproduction, water balance, feeding, metamorphosis, and sex attraction (Gäde and Goldworthy, 2003). Thus, disruption of these processes causes irreversible damage culminating in death. Unlike insecticides that are organic in nature and thus, xenobiotic peptide hormones do not cause harm to the environment, and are biodegradable. Thus, peptides that disrupt egg development and digestion in mosquitoes are good candidates for biodegradable mosquito control agents.

Factors that inhibit egg development (oostatic hormones and antigonadotropins) have been reported in cockroach, decapod crustaceans, and the house fly (Iwanov and Mescherskaya, 1935; Carlisle and Knowles, 1959; Adams et al., 1968; Kelly et al., 1984). In mosquitoes, the ovary secretes a humoral factor that inhibits yolk-deposition in less developed follicles (Else and Judson, 1972; Meola and Lea, 1972). These reports indicated that an ovarian factor synthesized by the ovary controls oogenesis in diverse insects. Borovsky (1985) demonstrated that the ovary contains a hormone that initially was named “oostatic hormone”. Injection of the hormone into decapitated and ovariectomized female mosquitoes directly stopped trypsin biosynthesis and blood digestion in the females gut and, therefore, the hormone affects the activity of female mosquito midgut cells that synthesize trypsin and not the ovary or endocrine tissues (Borovsky, 1988). The hormone was then named Trypsin Modulating Oostatic Factor (TMOF) and was purified sequenced and characterized by mass spectroscopy as an unblocked decapeptide having two close sequences (YDPAPPPPPP and DYPAPPPPPP; Borovsky et al., 1990). NMR analyses (Curto et al., 1993) suggested that the polyproline portion of TMOF in solution is a left-handed alpha helix. Oral applications and injections of TMOF was shown to inhibit serine proteases biosynthesis by disabling mosquitoes, cat flea, stable fly, house fly, and midge from digesting their blood meals (Borovsky et al., 1990, 1993). These results indicate that TMOF can traverse the gut epithelial cells into the hemolymph, bind TMOF gut receptor(s) with high affinity, and modulate trypsin biosynthesis (Borovsky et al., 1994). Based on these observations, Borovsky and Mahmood (1995), and Borovsky et al. (1989) suggested that TMOF could be used to control adult and larval mosquitoes.

To control mosquito larvae in the field we selected several potential microorganisms that are readily consumed by mosquito larvae and can produce TMOF in large quantities for field applications. In order to not pose a danger to the environment, these cells were heat inactivated before the release without inactivating TMOF.

Recent reports show that TMOF can be successfully expressed by *Chlorella desiccate* and in combination with *Bti cry* toxins in *Pichia pastoris* and without *Bti cry* toxins by *Saccharomyces cerevisiae* (Borovsky et al., 2010, 2011, 1994, 2018). These reports show that mosquito larvae readily consume these engineered cells and die. One advantage of using *P. pastoris* to express foreign genes is the presence of the alcohol oxidase promoters (P*_*AOX*_*_1_ and P*_*AOX*_*_2_). These promoters strongly depend on methanol but are also expressed in media containing other carbon sources like glycerol, glucose, and sorbitol (Thorpe et al., 1999). A second advantage for using these cells is the ease by which expression of foreign genes can be scaled up from shake-flask to high density fermenter cultures without the loss of yield. As a matter of fact, expression levels in shake flasks are usually lower than fermenter cultures make these cells ideal for industrial production of *tmf*A engineered cells.

This study expands the initial reports of engineered *P. pastoris* cells (Borovsky et al., 2010, 2011) and a book chapter (Borovsky, 2015) by describing a detailed biochemical and molecular biology analyses of the *tmf*A expressed in *P. pastoris*, detailed cloning strategies including Southern and Northern blot analyses, SDS PAGE of the recombinant *gfp-tmf*A expressed gene and high volume industrial production of *tmf*A by *P. pastoris* cells that have been approved by the EPA for use in the environment (Borovsky, 2007).

## Materials and Methods

### Genes Construction

All primers used in this study to construct *gfp-tmf*A, *tmf*A, and *gfp-*IEGR were synthesized by Gemini Biotech (FL, United States) and previously described for *S. cerevisiae* (Borovsky et al., 2018; Table 1). Two genes *gfp-tmf*A and *gfp-*IEGR were amplified by PCR using cycle 3 mutant *gfp* from the jellyfish *Aequorea Victoria* (accession number 1B9C_C), whereas a synthetic *tmf*A was prepared by annealing synthetic primers (Borovsky et al., 2018).

**TABLE 1:** Primers used for the genetic engineering of *P. pastoris* cells.

**Primers**	**Primer sequence (5′-3′)**	***t*_*m*_(°C)**
*tmf*A		
DB 192 (forward)	TCGAG**ATG**TATGATCCAGCACCT CCTCCTCCTCCTCCTTGAT	70
DB 193 (reverse)	CTAGA**TCA**AGGAGGAGGAGGAGGA GGAGGAGGTGCTGGATC ATACATC	68
*gfp-tmf*A		
DB 207 (forward)	AAGGTACC**ATG**GCTAGCAAAGGAGAAGAA	62
DB 209 (reverse)	TTTCTAGA**TCA**AGGAGGAGGAGGAGGA GGTGCTGGATCATA	68
	TCTACCTTCGATTTTGTAGAGCTCATCCAT	
*gfp*		
DB 207 (forward)	Sequence as above	
DB 230 (reverse)	TTTCTAGATTCATTTGTAGAGCTCATCCAT	57
*AOX*1		
DB 533 (forward)	AGATCTAACATCCAAAGACGA	51
DB534 (reverse)	CTCGTTTCGAATAATTAGTTG	48
RNA probe		
Nt 855–875 (forward)	GACTGGTTCCAATTGACAAGC	55
Nt 1160–1180 (reverse)	GCAAATGGCATTCTGACATCC	55

*Underlined sequences indicate restriction enzymes cleavage sites for the primers. DB 192 *Xho*I, DB 193 *Xba*I, DB 207 *Kpn*I, DB 209, and DB 230 *Xba*I. For RNA probe the position of the forward and reverse primers on the *AOX*1 is given in parenthesis. Nt = nucleotides. Bold letters denote start and stop signal.*

### Expression Vectors

All restriction enzymes used followed supplier recommendations (Gibco BRL, GA, United States). The constructed genes were digested with the appropriate restriction enzymes; *Kpn*I and *Xba*I for *gfp-tmf*A and plasmid pPICZB (Borovsky, 2015) was opened with the corresponding enzymes. For cloning *tmf*A, the vector was opened with *Xho*I and *Xba*I. Complete vector digestion was verified by agarose gel electrophoresis. Ligations were performed using T4 DNA ligase using overnight incubation at 14°C. Cloning into competent *Escherichia coli* cells was carried out by using heat shock at 42°C for 30 s. A control was included when an empty parental vector (without a gene inserted into its multiple cloning site) was cloned into competent *E. coli* cells. Transformants of *E. coli* InvαF’ were selected on Low Salt Luria-Bertani plates (1% Tryptone, 0.5% Yeast Extract, 0.5%NaCl, pH7.5, and 1.5% agar) containing 25 μg/ml Zeocin^TM^ (Invitrogen, CA, United States). Zeocin^TM^-resistant transformants were grown on Low Salt LB medium (1% Tryptone, 0.5% Yeast Extract, 0.5%NaCl, and pH7.5) with 25 μg/ml Zeocin^TM^ overnight at 37°C. Plasmids were extracted and purified using QIAprep Spin Miniprep kit (Qiagen, CA, United States). Screening of recombinants was done by restriction enzyme and PCR analyses. Plasmids that contained inserts were sequenced by the dideoxynucleotide chain termination method (Sanger et al., 1977) with [α^35^S]dATP and the enzyme T7Sequenase (version 2.0; US Biochemicals, OH; Tabor and Richardson, 1987) or with ABI PRISM^®^BigDye^TM^ Terminator Cycle Sequencing Ready Reaction Kit (PE Biosystems, MA, United States). Removal of excess BigDye^TM^ terminators from completed DNA sequencing reactions was done using DyeEx kit (Qiagen, CA, United States) and DNA was analyzed using Applied Biosystems Model 377 DNA sequencer (Perkin Elmer, CA, United States).

### Cloning Into *Pichia pastoris* and Screening for Multi-Copy Recombinants

Competent *P. pastoris* KM71 or KM71H cells were prepared using the *Pichia.*EasyComp^TM^ transformation kit (Invitrogen, CA, United States) and aliquots (50 μl) were stored at −80°C. Transformation was performed following the LiAc/SS-DNA/PEG procedure (Gietz et al., 1995) using *Pichia.*Easycomp transformation kit (Invitrogen, CA, United States). Prior to transformation, the pPICZB vectors (Borovsky, 2015) were linearized with *BstXI* to facilitate homologous recombination at the *AOX*1 promotor locus. Transformants were selected on YPDS plates (1%Yeast Extract, 2% Peptone, 2% Dextrose, 1 M Sorbitol, and 2% Agar) with 100 μg/ml Zeocin^TM^ at 30°C. Transformants selected on 100 μg/ml Zeocin plates were further tested on YPDS plates with increased concentrations of Zeocin^TM^ (200 to 3000 μg/ml). The plates were incubated for several days at 30°C. Colonies that grew on the highest concentrations of Zeocin were selected.

### Gene Expression by *Pichia pastoris* Cells (Shake Flask Fermentation)

Single colonies of the multi-copy transformants that were selected with Zeocin (100 and 3000 μg/ml) for single and multiple copies of *tmf*A on YPDS plates (above) were removed and grown at 30°C in 10 ml of MGYH (minimal glycerol medium + histidine; 1.34% yeast nitrogen base with ammonium sulfate without amino acids, 1% glycerol, 4 × 10^–5^ biotin, and 0.004% histidine) overnight in a shaking incubator at 300 rpm or without histidine when KM71H cells were used. In the morning, MGYH (200 ml) was inoculated with the overnight culture and grown at 30°C until the culture reaches an (Optical Density) OD_600_ of 4. The cells were harvested by centrifugation and the pellet resuspended in 40 ml of MMH (minimal methanol medium +histidine) 1.34% yeast nitrogen base with ammonium sulfate without amino acids, 4 × 10^–5^% biotin, 0.004% histidine, and 0.5% methanol). The cultures were then stimulated for up to 144 h and every 24 h an aliquot (10 ml) was taken and frozen at −20°C until further analysis. To maintain induction, at 24 h intervals, 100% methanol was added to a final concentration of 0.5%.

### Large Scale Fermentation

Large scale fermentation (150 L) of *P. pastoris* cells KM71 and KM71H engineered with *tmf*A and *gfp-tmf*A were done by BRI at NRC (Canada). Transformed cells were grown at 30°C with agitation rate of 400–1000 rpm, airflow of 7–14 L/min, pressure of 0.05 bar, and pH control at 6.0. During growth on glycerol ammonium hydroxide was used and during the induction phase phosphoric acid was used. Dissolved oxygen was at 20% saturation. At induction time 15 L of 5X BMGY (complex medium) medium without glycerol but with methanol (0.7% v/v) and 50% of the required phosphate buffer was added to prevent precipitation and to minimize potential nutrients starvation during induction. Additional nutrients were added at 16 and 40 h after induction. Fluorescence of cells engineered with *gfp-tmf*A was monitored during the fermentation and fluorescence reached a maximum at 144 h. Methanol concentration was maintained between 0.5 and 0.7% and monitored by GC. Samples were removed at different intervals during the fermentation (0, 21, 45, 69, 78, 93, 117, and 142 h) heated at 75°C for 3 h or subjected to large scale drying, using an oven blower in which the samples entered at 200°C and left at 85°C. The heat treatment of the cells weakened the cell wall inactivated the cells and allowed larvae to digest the yeast cells more efficiently thus, obtaining more TMOF.

### ELISA (Enzyme Linked Immunoadsorbant Assay)

At intervals during the fermentation 1.5 × 10^8^ cells/ml were collected and centrifuged down, supernatants discarded and to each pellet of Y-PER (400 μl) Yeast Protein Extraction Reagent (Pierce, IL) was added and incubated for 20 min at room temperature. Cells were then broken using Fast Prep Instrument FP120 (BIO 101, CA) at speed 6 for 20 s with glass beads. Broken cells were spun down for 5 min at 14,000 RPM at room temperature and the supernatant kept. The glass beads were then washed with 400 μl PBS buffer, pH 7.2, and added to the supernatant and stored at −20°C.

Enzyme Linked Immunoadsorbant Assay was determined using Reacti-Bind^TM^ Maleic Anhydride Activated Polystyrene Plates from Pierce (Borovsky et al., 1992, 2010). Briefly, Samples with TMOF were diluted 1:400, 1:800,1:1000, and 1:1600-fold were bound to the plate wells in PBS (100 μl), pH7.2 (0.1 M Phosphate,0.15 M NaCl) overnight by gentle shaking. The wells were decanted and to each well blocking solution (300 μl) pH 7.5 containing 50 mM Tris–HCl, 0.15 M NaCl, 0.05%Tween^®^ 20 and 0.3% BSA was added and incubated for 1 h at room temperature. The solutions were decanted from the wells and to each well 100 μl of the anti-peptide antibody (anti-TMOF) diluted in dilution buffer (PBS, 0.1% BSA, and Tween^®^ 20 0.05%) was added. The plates were then incubated for 1 h with gentle shaking. The wells were then washed three times with washing buffer (50 mM Tris–HCl, 0.15 M NaCl, 0.05%Tween^®^ 20, 2% polyvinylpyrolidone, 0.1% BSA, and pH7.4). After the third wash 100 μl of goat anti rabbit antibody linked to alkaline phosphatase that was diluted in dilution buffer was added. The wells were incubated for 1 h with gentle shaking. After three washes, 200 μl of alkaline phosphatase liquid substrate from Sigma was added for detection. After 30 min of incubation the plates were read at 405 nm in a Microplate reader from Bio-Tex. A calibration curve was constructed with known concentrations of the peptide TMOF.

### Southern Blot Analysis

*Pichia pastoris* genomic DNA was isolated using a fast DNA kit (BIO 101, CA, United States) or a DNeasy tissue kit (Quiagen, CA, United States). For the fast DNA kit, yeast cells from each clone (1.5 × 10^8^ to 3 × 10^8^ cells) were broken in 2 ml tubes containing 0.25 inch Sphere and Garnet Matrix and 1 ml of CLS-Y (cell lysis/DNA solubilization solution) using a FastPrep instrument (FP120, BIO 101, CA, United States). Broken cells were centrifuged, and DNA was bound to DNA binding matrix solution. The bound DNA-matrix was then centrifuged, the pellet washed with salt ethanol wash solution and the DNA eluted from the matrix with DNA elution solution (BIO 101, CA, United States), the solution centrifuged, and the supernatant collected and stored at −20°C. For the Qiagen DNeasy Tissue kit yeast cells (3 × 10^7^ cells) were centrifuged, and the pellet resuspended in PBS (200 μl), and AL buffer (200 μl; Qiagen, CA, United States). The suspended cells were broken with glass beads for 20 s using FastPrep (FP125, BIO101 Savant, CA, United States). To the broken cells Proteinase K was added and the homogenate incubated at 70°C for 10 min. After incubation, the broken cells homogenate was centrifuged for 5 min at 14,000 rpm and the supernatant transferred to a fresh tube and ethanol (200 μl) was added and the mixture adsorbed onto DNeasy spin columns and genomic DNA was eluted after several washes following manufacturer’s guideline and stored at −20°C.

A 942 bp *AOX*1 probe was amplified by PCR using pPICZB and the following primers: DB 533 (forward), 5′- AGATCTAACATCCAAAGACGA-3′ and DB534 (reverse), 5′-CTCGTTTCGAATAATTAGTTG-3′. A 700 bp *gfp* probe was amplified by PCR using pYDB2*gfp* and primers, DB207, and DB230 (Borovsky et al., 2018). The probes were labeled with [α^32^P] dCTP using Rediprime^TM^II labeling system (Amersham Pharmacia Biotech, United Kingdom) and the labeled probes purified using Qiaquick PCR column (Qiagen, CA, United States). The 46 bp TMOF oligonucleotide (DB192; Borovsky et al., 2018) was labeled with [γ^32^P] dATP using RTS T4 Kinase (GibcoBRL, MD). Ambion SouthernMax^TM^ kit was used for Southern analysis (Ambion, TX, United States). *P. pastoris* genomic DNA, prepared using a FastDNA^®^ Kit (BIO101), was digested with 5 U of *Eco*RI (Gibco-BRL, MD). DNA digests (10 μg per lane) were separated on a 0.8% agarose gel and transferred to BrightStar-Plus positively charged nylon membrane (Ambion, TX, United States) using a Turboblotter (Schleister & Schuell, Germany). The membrane was blocked and hybridized with a purified denatured radioactive labeled DNA probe or a radioactive labeled oligonucleotide. Prehybridization and hybridization was performed at 42°C in rotating hybridization bottles in a hybridization oven (Enprotech, OH, United States). After washing, the membrane was wrapped in plastic foil and exposed to an X-ray film. Between hybridizing with each probe, the membrane was washed with a boiling 0.1% SDS in sterile water solution for 15 min to strip the label.

### Northern Blot Analysis

RNA from transformed *P. pastoris* cells (1 mL) were isolated using TRIzol (Gibco BRL, CA, United States). The cells were broken for 20 s using speed of 4 in a Fast Prep Instrument (Savant, CA, United States) using glass beads following manufacturer instructions. The RNA was precipitated with isopropanol (0.5 mL) and the pellet washed with 75% ethanol and dried at room temperature for 10 min. The dried RNA was redissolved in 100 mL of DEPC (diethyl pyrocarbonate) treated sterile water at 57°C for 10 min and the concentration was determined at 260 nm using GeneQuant spectrophotometer (Amersham Pharmacia, United Kingdom). To detect the RNA transcript a probe (360 bp) was prepared by PCR (Borovsky et al., 2018) using *AOX*1 as a template and forward and reverse primer pair (5′-GACTGGTTCCAATTGACAAGC-3′ and 5′-GCAAATGGCATTCTGACATCC-3′), respectively.

RNA transcript levels in engineered *P. pastoris* cells were determined according to Sambrook et al. (1989) using NorthernMax kit (Ambion, TX, United States). RNA aliquots (3 μg) and standards were separated by gel electrophoresis (80 V for 1.5 h) on 1% denatured agarose gels according to manufacturer’s instructions (Ambion, TX, United States). Samples were mixed with formaldehyde loading dye and incubated for 15 min at 65°C prior to electrophoresis. Following electrophoresis, the RNA was transferred for 3 h from the gels unto BrightStar-Plus positively charged nylon membranes using transfer buffer (Ambion, TX, United States), and a Turboblotter (Schleister & Schull, Keene, NH, United States). The RNA was cross linked to membranes at 120°C for 15 min and the membranes were stored at −20°C. Standards were visualized by methylene blue (Sambrook et al., 1989). The membranes were prehybridized for 4 h in ULTRAhyb (20 ml; Ambion, TX, United States) and hybridized overnight with purified denatured [^32^P]DNA probe. Prehybridization, hybridization and washing steps were done at 42°C in rotating hybridization bottles in an oven (Enprotech, MA, United States). After low and high stringency washes (Ambion, TX, United States) the membranes were wrapped in plastic film to prevent drying and exposed to X-ray film for 24 to 72 h at −80°C.

To allow re-probing of the membranes with an actin probe (300 bp) based on *P. pastoris act*1 transcript (accession number AF216956) was amplified by PCR using primer pair (forward) 5′- TTAGTTATCGACAATGGTT-3′ (*t*_*m*_ 55°C) and (reverse) 5′-CCTCAGTCAAAAGAACTG-3′ (*t*_*m*_ 57°C), the membrane was incubated with SDS (0.1%) solution that was preheated to 100°C. The incubation mixture was cooled down to room temperature, the membrane removed, and scanned for radioactivity with a hand-held Geiger counter. The procedure was repeated until no radioactivity was detected. The hybridization procedure and the washings were repeated as mentioned above and the X-ray film was exposed for 24 to 72 h at −80°C (Borovsky et al., 2016).

The Northern blots were scanned using Sapphire Biomolecular Imager (azure biosystems) using 3 wavelengths (488, 520, and 658 nm) and the scanned *tmf*A transcript bands were divided by the scanned *act* transcript bands that were used as a reference gene. The ratios were then plotted against different fermentation times and were used to compare the *tmf*A transcripts of low copy and high copy engineered *P. pastoris-tmf*A cells.

### 3D Model

**FIGURE 1:**
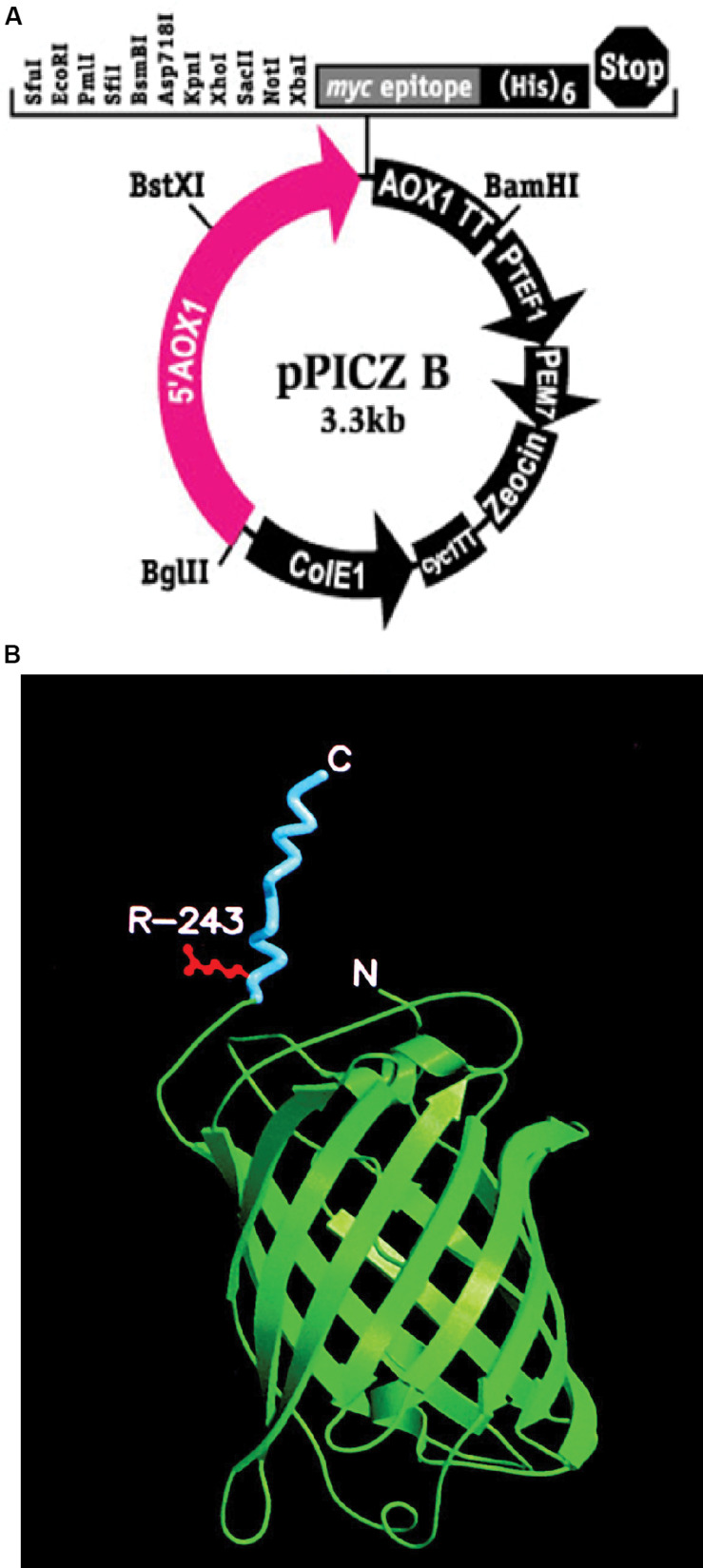
**(A)** pPICZB plasmid that was used to transform KM71 and KM71H cells. The plasmid multiple cloning site is shown including the positions of the restriction enzyme pairs *Xba*I and *Kpn*I and *Xba*I and *Xho*I that were used to clone *gfp*-*tm*fA and *tmf*A, respectively. Shown also is the P*_*AOX*_*_1_ and the restriction enzyme position for *Bst*XI used to open the plasmid for homologous recombination. The arrows indicate the direction of replication. **(B)** A 3D model of the recombinant fusion protein GFP-TMOF including R243 where mosquito larva cleaves the fusion protein in the gut releasing TMOF. The carboxyl and amino ends are denoted as C and N, respectively. TMOF (blue) assumes a left handed conformation and GFP green a barrel conformation made of β-pleated sheets.

A three-dimensional ribbon drawing model of GFP-TMOF recombinant protein was built (Figure 1B) using SYBYL molecular modeling software (v. 6.3) and converted into a ribbon representation by the program Molecular Molscript (Kraulis, 1991; Borovsky et al., 1996; Yan et al., 1999) and is based on X-ray diffraction conformation for GFP (PDB 1EMA) and NMR presentation of TMOF (Curto et al., 1993).

### Monitoring GFP-TMOF Expression

Expression of the GFP-TMOF in *P. pastoris* engineered cells was monitored using short wave UV-254 nm Lamp (model UVS-11, Ultra-Violet products, CA, United States) in a dark room. Fluorescent engineered cells were photographed and compared with the same cells that were radiated with normal light.

### SDS PAGE Analysis

Engineered *gfp-tmf*A *P. pastoris* (1.5 × 10^8^ cells) were pelleted at 14,000 rpm for 5 min and lysed by vortexing in 50 mM Tris–HCl, pH 7.9, 2% SDS, 5% β-mercaptoethanol using glass beads for 30 min at 4°C. The mixture was heated for 5 min at 90°C, centrifuged at 14,000 rpm for 5 min at room temperature and supernatants collected. Aliquots (80 μl) were removed and mixed with 20 μl of sample buffer [0.5 M Tris–HCl, pH 6.8, glycerol 10% (v/v), 25% β-mercaptoethanol, and 0.05% Bromophenol blue]. The extracted proteins were separated by SDS-PAGE on a slab poly acrylamide gel (Laemmli, 1970) and stained with Coomassie Blue R-250 and distained in MeOH acetic acid for 18 h. Distained gels were then dried using a gel drier at 70°C (BioRad, CA, United States).

### Larvicidal Activity of the Engineered *P. pastoris* Cells

*Aedes aegypti* eggs were placed in deionized water supplemented with live yeast extract to induce egg hatching. Single newly hatched larvae were added to separated wells of 48 microtiter plates at room temperature. Each well contained sterile water (1 ml) and 2 × 10^7^ engineered yeast cells. The yeast cells were washed three times by centrifugation before feeding them to larvae to get rid of the culture medium that the cells were growing in. Cells were then heat treated at 80°C for 3 h and after shake flask fermentation, or for large scale fermentation dried in an oven at 200°C when cell entered the oven and at 85° upon exit. Larval growth and mortalities were monitored daily up to 12 days. For large scale testing, larvae (100 per group) were tested in 200 ml water with different concentrations of recombinant *P. pastoris* KM71H-*tmf*A heat treated dried yeast cells in an oven as mentioned above.

### Data Analysis

Data are expressed as means of 3 determinations ± SEM. Graphs were plotted using GraphPad Prism 5.0 (GraphPad, CA, United States).

## Results

### Cloning and Expressing Genes in *P. pastoris*

**FIGURE 2:**
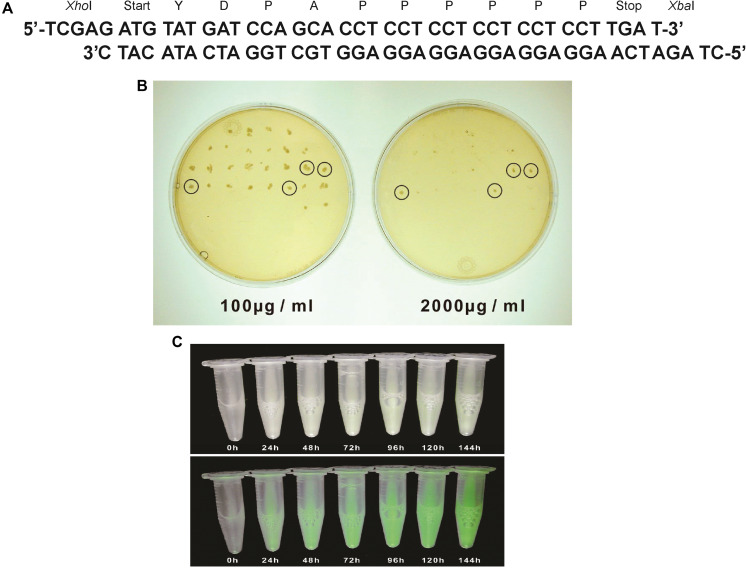
**(A)** A synthetic *tmf*A made up of 2 oligonucleotides that were annealed and used for cloning *tmf*A into pPICZB. The two restriction site positions *Xho*I and *Xba*I and start and stop signals and the amino acids sequence of TMOF are also shown. **(B)** YPDS agar plates to select transformed KM71H-*tmf*A for high copy number and low copy number on Zeocin (100 μg/ml and 1000 μg/ml, respectively). Only 4 colonies survived in the presence of high Zeocin concentration, whereas many colonies survived at low Zeocin concentration. **(C)** KM71-*gfp-tmf*A engineered cells that were fermented by shake flask for (0–144 h). Cells that were exposed to regular light (upper panel) did not fluoresce, whereas cells that were exposed to UV light (lower panel) increased in fluorescence as the fermentation progressed.

*Pichia pastoris* cells were transformed using a multi copy integrating plasmid pPICZB (Invitrogen, CA, United States; Figure 1A). The plasmid has a multiple cloning site and a strong alcohol oxidase promoter (P*_*AOX*_*_1_) allowing selection using Zeocin and ColE1 origin allowing replication and selection in *E. coli.* Two genes *tmf*A and *gfp-tmf*A were amplified by PCR using primer pairs DB 192 and DB 193 for *tmf*A and DB 207 and DB 209 for *gfp-tmf*A (Table 1). The *tmf*A and *gfp-tmf*A amplicons were digested with *Xho*I and *Xba*I and *Kpn*I and *Xba*I, respectively, to insert the two genes into the multiple cloning site of pPICZB (Figure 1A). The *gfp-tmf*A codes a Green Fluorescent protein TMOF fusion protein with an IEGR trypsin cleavage site between GFP and TMOF at R243 (Figure 1B). The TMOF nucleotides sequence is based on the amino acid sequence of the decapeptide (Figure 2A). After ligation of *tmf*A and *gfp-tmf*A into pPICZB the inserts were sequenced, the plasmids were linearized with *Bst*XI and *P. pastoris* strains KM71 (*arg4aox1*Δ::*ARG4,his4*), and KM71H (*arg4aox1*Δ::*ARG4*) were transformed by homologous recombination at the P*_*AOX*_*_1_ locus (Figure 1A) and screened for multiple insertions using Zeocin (Figure 2B). KM71 and KM71H strain carry Mut^*s*^ with a phenotype that utilizes methanol slowly. They also have a mutation at the *aox*1 locus in which the *AOX*I gene is largely deleted and replaced with *S. cerevisiae ARG4 gene* and wild type *AOX2* gene that metabolizes methanol slower and therefore the cells grow slow in medium containing methanol. After transformation the transformants were screened for multiple insertions by selecting the colonies on increasing concentrations of Zeocin because there is a strong correlation between drug resistance and copy number. Colonies were selected on 2000 μg/ml from the KM71-*tmf*A (Figure 2B) and KM71-*gfp-tmf*A transformed cells. Several colonies were also selected from KM71H-*tmf*A transformed cells on 3000 μg/ml Zeocin and a colony was selected for comparison on 100 μg/ml Zeocin. The cells that were selected on Zeocin were fermented in methanol using shake flask and large scale (150 L) fermentations for up to 144 h at 30°C and aliquots (10 ml) were removed for ELISA at intervals and were also fed to mosquito larvae. *P. pastoris* cells KM71-*gfp-tmf*A that were fermented in shake flask (0–144) were monitored with UV light. In the absence of UV light, the cells did not fluoresce and increase in fluorescence was observed with increase time of the fermentation reaching a peak between 72 to144 h (Figure 2C, upper and lower panels).

### Copy Number of *gfp* and *gfp-tmf*A

**FIGURE 3:**
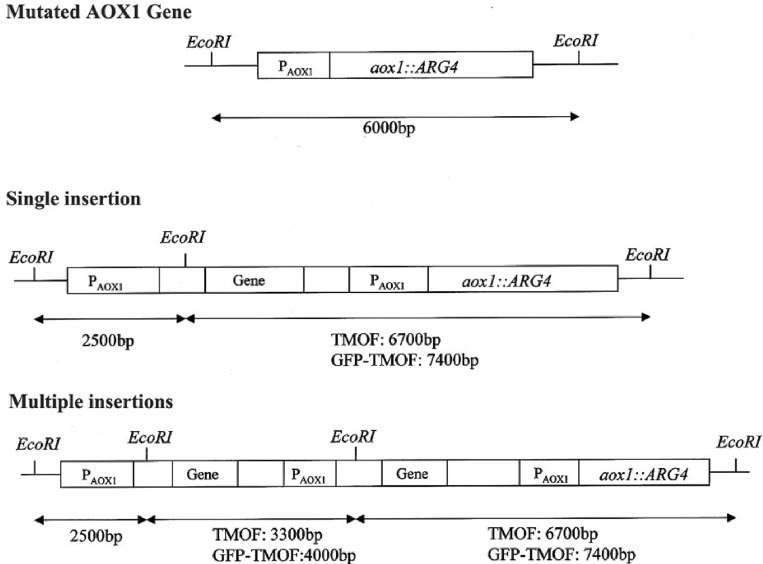
Genetic map of KM71 and KM71H cells with mutation at the *aox*1 and its promoter showing the DNA size and *Eco*RI restriction sites. A genetic map after a single insertion of *tmf*A and *gfp-tmf*A including *Eco*RI restriction sites and expected restriction fragments after *Eco*RI digestion. A genetic map of multiple insertion of *tmf*A and *gfp-tmf*A including additional *Eco*RI sites and restriction fragments.

**FIGURE 4:**
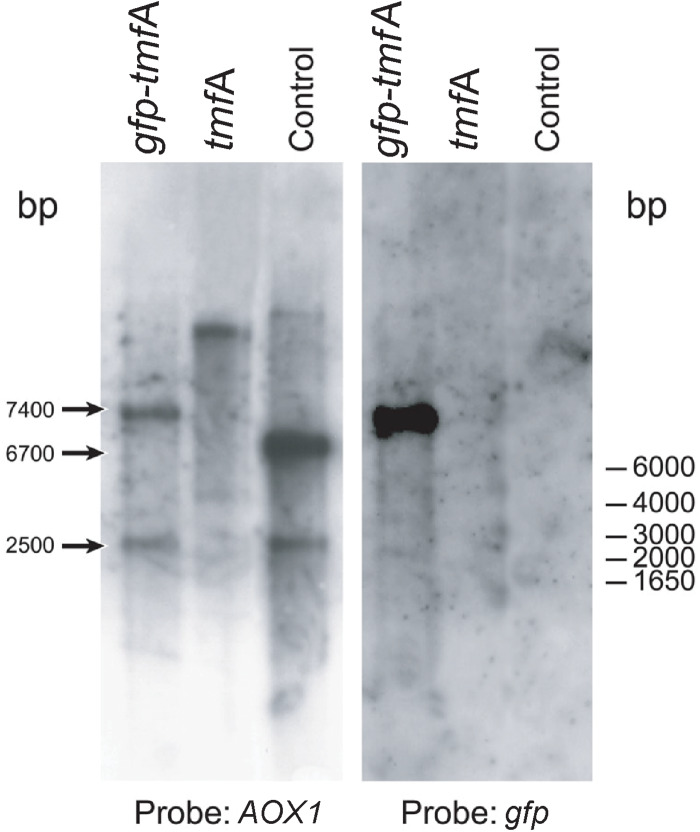
Southern blot analyses of genomic DNA of transformed KM71-*tmf*A and KM71-*gfp-tmf*A cells. The blots were probed with *AOX*1 (left blot) and *gfp* (right blot) generated by PCR (Table 1). The size of DNA markers is given on the right and the sizes of the digested DNA bands is indicated with arrows on the left. The Southern blot was repeated a second time showing the same results.

**FIGURE 5:**
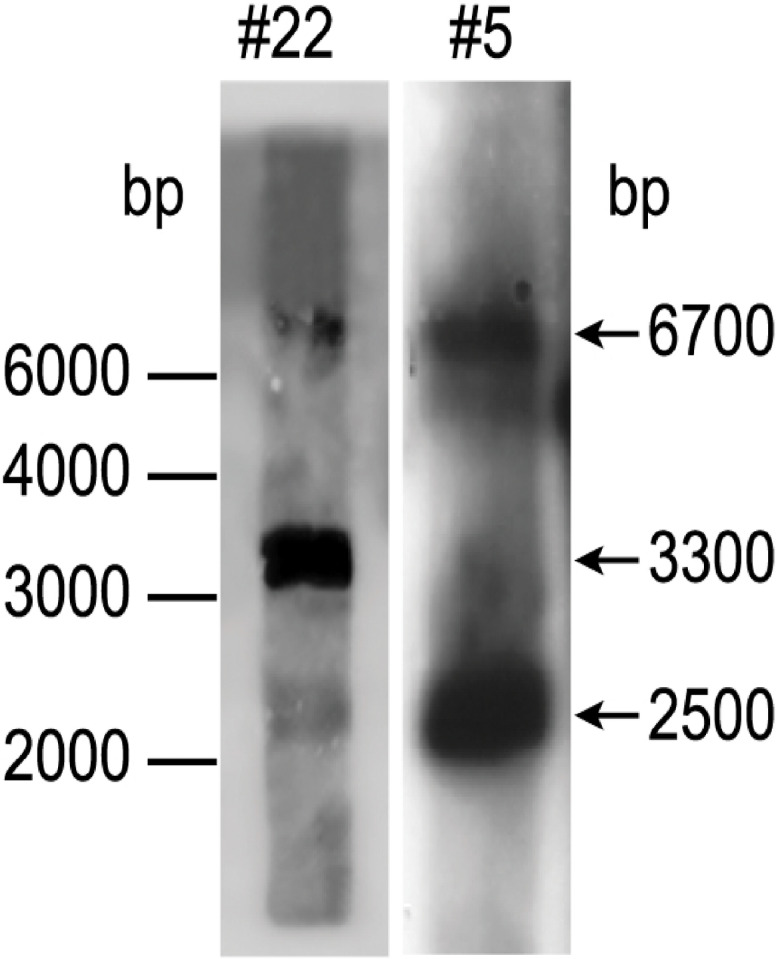
Southern blot analysis of *P. pastoris* KM71-*tmf*A colonies #22 and #5. DNA was extracted and digested with *Eco*RI and analyzed by Southern blotting using *aox*1 probe. The size of the DNA markers is on the left and the sizes of the digested DNA bands is indicated with arrows on the right. The Southern blot was repeated a second time showing the same results.

To find out how many *tmf*A and *gfp-tmf*A genes were incorporated into the genome of *P. pastoris* after homologous recombination, three genetic maps of *P. pastoris* were constructed. Wild type with mutated *AOX*1 gene is about 6000 bp if it is cut with *Eco*RI (Figure 3; Ellis et al., 1985). A single insertion of *tmf*A or *gfp-tmf*A would generate an additional *Eco*RI restriction site of 2500 bp and 6700 bp or 2500 bp and 7400 bp bands, respectively (Figure 3, single insertion), whereas double insertion of *tmf*A or *gfp-tmf*A will generate 3300 bp and 6700 bp or 4000 bp and 7400 bp bands, respectively (Figure 3, double insertions). Southern blotting analysis of genomic DNA of KM71 cells that were transformed with *gfp-tmf*A and *tmf*A and were digested with *Eco*RI identified two DNA bands (7400 and 2500 bp) for *gfp-tmf*A and one for *tmf*A (9200 bp) when probed with *AOX*1. The larger DNA band of 9200 bp indicate that the *Eco*RI restriction site was lost during the homologous recombination (Figures 3, 4 left blot; Supplementary Material 1). Two expected bands of 2500 bp and 6700 bp were detected in *P. pastoris* cells that were transformed with an empty pPICZB plasmid (Figure 4 left blot, control; Supplementary Material 2). Probing the blot with *gfp* probe after the *AOX*1 probe was stripped from the blot identified a single band of 7400 bp when recombinant cells with *gfp-tmf*A were probed with *gfp* probe, and no DNA band was detected when recombinant cells carrying *tmf*A or transformed with empty plasmid were probed with the *gfp* probe (Figure 4, right blot). These results indicate that the KM71 cells that were transformed by homologous recombination have a single insertion of *gfp-tmf*A and *tmf*A. Transforming KM71H, however, with *tmf*A and analyzing two of the transformed colonies (#22 and #5) using Southern blot analysis and *AOX*1 probe show that in both cases the 3 expected bands of 2500, 3300, and 6700 bp are found (Figure 5), however, the ratio of the 3300 bp band to the 6700 bp band in colony #22 is 10 times higher as judged by the intensity of the bands (determined with KODAK EDAS 290 image analyzer) indicating that the transformed cells have at least 10 copies of *tmf*A (Figure 5). On the other hand, the intensities of the 3300 bp and the 6700 bp bands of colony #5 are equal (determined as above) indicating that this colony incorporated a single *tmf*A (Figure 5). The Southern blot analysis were repeated twice with similar results.

### Transcript Analysis of KM71H-*tmf*A Cells

**FIGURE 6:**
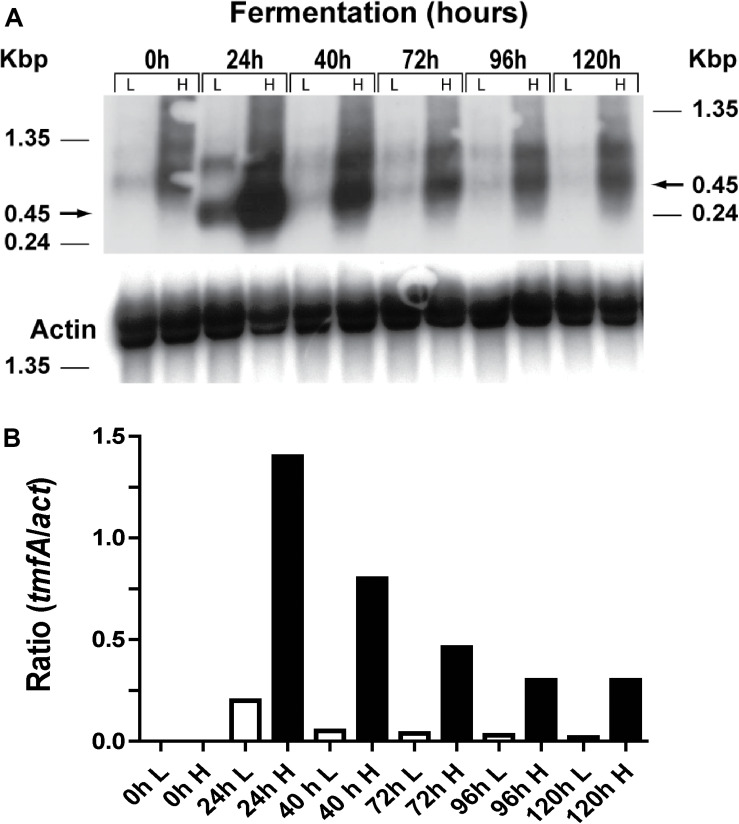
Northern blot analysis of KM71H-*gfp-tmf*A cells (#5 and #22, low and high copy, respectively) that were fermented by shake flask for 0–120 h. **(A)** Total RNA (3 μg/lane) was analyzed using a specific probe amplified by PCR (Table 1). The size of the RNA markers is given on the left and the *tmf*A transcript (450 bp) is shown by an arrow on the right. **(B)** Scanned *tmf*A transcript blots (see a above) after different hours of the fermentation (0–120 h) of high and low copy colonies engineered with *tmf*A were compared with *act* transcript as a reference gene and the transcript ratios of *tmfA/act* were compared for cell that were engineered with 10 and 1 *tmf*A genes, respectively. White bars represent cells expressing a single *tmf*A and black bars cells expressing 10 *tmf*A genes. L = low copy colony #5 and H = high copy colony #22. The Northern blot analyses were repeated two times showing the same results.

Two clones (#5 and #22) that integrated one and 10 copies of *tmf*A, respectively (Figure 5) were analyzed by Northern blot analysis by removing samples at different intervals during shake flask methanol fermentation of the cells (0, 24, 48, 72, 96, and 120 h; Figure 6A and Supplementary Material 3). A *tmf*A transcript of 450 bp was detected at 24 h and no message was detected before the methanol induction time (0 h) in cells with low (L) and high copy number (1 and 10 copies, respectively; Figure 6A). At different times during the fermentations the *tmf*A/*act* ratios of cells carrying 10 *tmf*A copies were much higher than cells carrying one copy (Figure 6B). When the *tmf*A/*act* ratios of cells carrying 10 copies were compared with the ratios of cells that carried one copy a 7-fold difference was found at 24 h, 16-fold difference at 40 h, 11-fold difference at 72 h, 10-fold higher at 96 h, and 15-fold higher at 120 h (Figure 6B). As the fermentation proceeded the intensity of the blots dropped, however, the ratios of *tmfA/act* transcripts in cells carrying 10 copies as compared with cell carrying a single copy were close. These results show that engineered cells with 10 copies of *tmf*A expressed more transcript throughout the fermentation than cells with one *tmf*A copy. The *act* a house keeping gene was used as a reference gene for the analysis of the Northern blot (Figure 6B) and to show a uniform transfer of the *tmf*A transcript in all the lanes (Figure 6A). The experiment was repeated twice with similar results.

### Detection of GFP-TMOF by SDS PAGE

**FIGURE 7:**
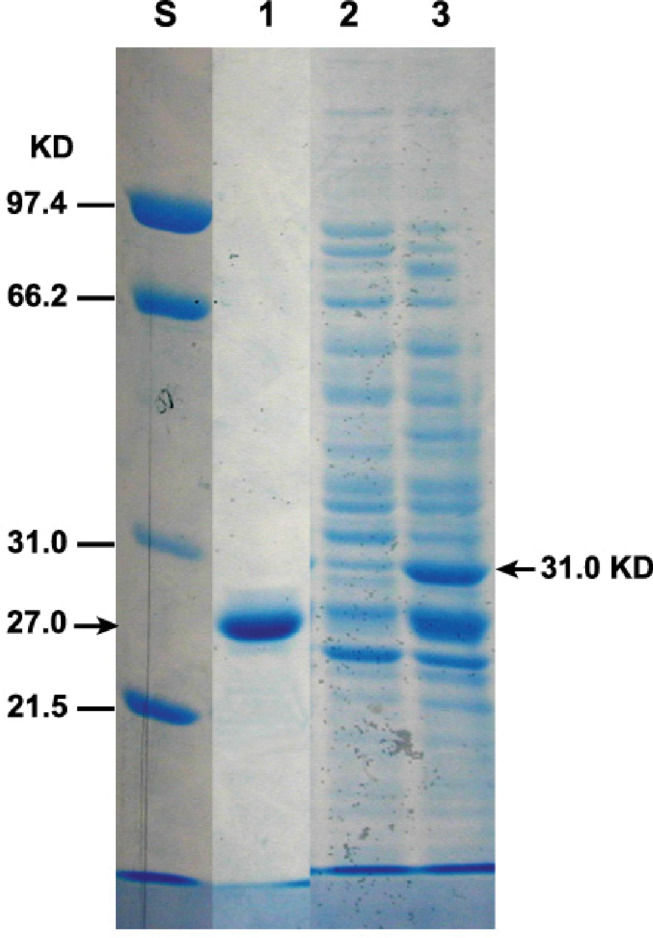
SDS-PAGE of KM71-*gfp-tmf*A transformed cells. Each lane contains equal amount of proteins extracted from equal number of cells (1.6 × 10^7^) and separated by SDS-PAGE. Protein were stained with Coomassie Blue. Marker protein of known size were run on the left lane (S). Lane 1 standard GFP (27.6 kDa), lane 2 protein extracted from non-stimulated cells, Lane 3 cells stimulated with 0.5% methanol for 93 h. GFP-TMOF expected protein band (31 kDa) is indicated with an arrow on the right.

To detect the expressed GFP-TMOF fusion protein, *P. pastoris* cells that were transformed with KM71-*gfp-tmf*A were fermented for 93 h or immediately used (0 h). The cells were broken with glass beads in the presence of SDS and proteins were separated by SDS-PAGE and the gel stained with Coomassie Blue. Two heavily stained bands of 31 kDa and a second band that has a similar mobility with the GFP standard were detected (Figure 7, lanes 3, and 1). The protein band that ran at 31 kDa corresponds to the GFP-TMOF fusion protein, whereas the protein band that ran below the 31 kDa band with similar mobility of the GFP standard may be protease digested GFT-TMOF at the IEGR site (Figure 1B) during the glass beads extraction of the recombinant protein.

### Detection of TMOF by ELISA

Although our Northern blot analysis (see above) clearly indicated that the amount of *tmf*A transcript is at least 2-fold higher if the intensities of the bands are observed and at 7 to 16-fold higher if the transcript bands are normalized with *act* as a reference gene (Figures 6A,B) it does not necessarily mean that the amount of TMOF expressed by the high copy transformed cells is different than cell expressing one copy. To find out if high expression of *tmf*A transcript results in higher amount of TMOF, the engineered *P. pastoris* KM71 cells, proteins were extracted from cells at 96 h when KM71-*gfp-tmf*A and KM71-*tmf*A cells were fermented by shake flask fermentation and analyzed by ELISA (Borovsky et al., 1992). The amount of GFP-TMOF after 96 h fermentation of 3 × 10^8^ cells was 10.2 ± 1 (μg ± SEM). KM71-*tmf*A 3 × 10^8^ engineered cells, produced 12 ± 1.4 (μg ± SEM). The same number of KM71H-*tmf*A cells (10 insertions, clone #22, and Figure 5) that were fermented by industrial fermenters produced about 4 to 10-fold more TMOF after 120 h using 5 to 150 L fermenters (Table 2). We also purified the extracted proteins by HPLC from 3 × 10^8^ cells (Borovsky et al., 1990, 1993). Even though, high losses occurred during the lengthy purification steps we showed that after 120 h of shake flask fermentation of engineered KM71H-*tmf*A cells 136 ± 13 (ng ± SEM; *n* = 3) could be detected proving that the engineered cells synthesize TMOF.

**TABLE 2:** Synthesis of GFP-TMOF and TMOF using shake flask and large volume fermentations of *P. pastoris* KM71-*gfp-tmf*A and KM71-*tmf*A cells.

**Fermentation (h)**	**N**	**μg/3 × 10^8^ cells**	**Size (L)**
**a. Shake flask**			
*KM71-gfp-tmfA*			
96	3	10.2 ± 1	0.4
*KM71-tmfA*			
144	6	12 ± 1.4	0.4
**b. Industrial fermentation**			
*KM71H-tmfA (colony #22)*			
120	3	44 ± 6	5
120	3	101 ± 14	5
120	3	19.2 ± 5	20
120	3	32.4 ± 10	150

**P. pastoris* cells transformed with KM71-*gfp-tmf*A, KM71-*tmf*A (one copy), and KM71H-*tmf*A (10-copies) were fermented in a shake flask or in large volumes using industrial fermenters. Aliquots (3 × 10^8^ cells) were removed at different times and assayed for TMOF using ELISA (Borovsky et al., 1992). Industrial fermented KM71H-*tmf*A (clone #22) cells were dried in an oven entry temperature 210°C and exit temperature 85°C. Results are average of 3 to 6 determinations ± SEM.*

### Biological Activity of *P. pastoris* Engineered Cells

#### Shake Flask Fermentations

**FIGURE 8:**
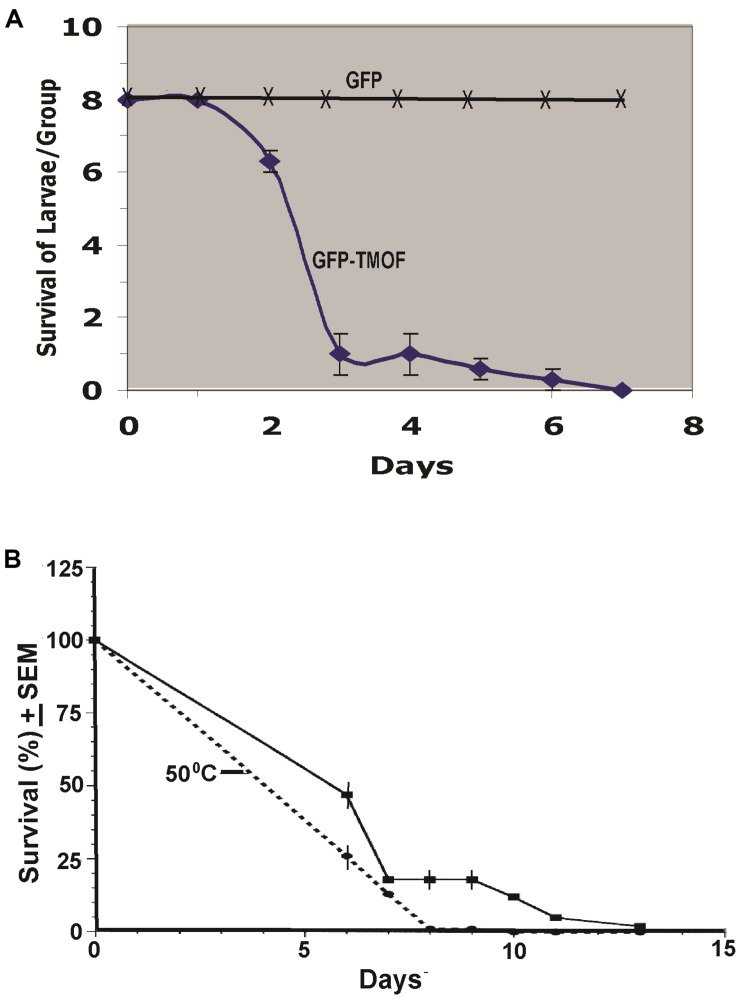
**(A)** Feeding first instar *Ae. aegypti* larvae KM71-*gfp-tmf*A and KM71H-*gfp* (control) cells after shake flask fermentation and heat treatment (80°C for 3 h). Three groups (8 larvae per group) were fed the engineered cells (2 × 10^7^ cells) in 48 well plate containing one larva in 1 ml water per well for 7 days. **(B)** Comparison between KM71-*tmf*A cells that were heated at 55°C for 3 h (broken line) and cells that were not heated (solid line) and were fed to 6 groups of 100 larvae per 200 ml for 13 days. Mortalities were followed daily and the results are expressed as means ± SEM.

Engineered *P. pastoris* cells KM71-*gfp-tmf*A were fermented in shake flask and heated to 80°C for 3 h. After the heat treatment the cells were washed with distilled water (3 times) and the washed cells were fed to first instar *Ae. aegypti* larvae in 48 well plate (1 larva per well) containing engineered *P. pastoris* (2 × 10^7^ cells) in 1 ml of distilled water. Three groups of 8 larvae were fed the recombinant cells for 12 days and mortality was daily monitored. 88% of the larvae died within 3 days, whereas larvae that that were fed *P. pastoris* KM71-*gfp* without *tmf*A all survived, controls (Figure 8A). In a second experiment *P. pastoris* KM71-*tmf*A (2 × 10^7^ cells/ml) were heated at 55°C for 3 h and compared with control cells that were not heated. Six groups of 100 *Ae. aegypti* first instar larvae were each fed in pans containing 200 ml distilled water. The larvae that were fed heated cells all died within 8 days whereas the larvae that were fed nonheated cells all died at 13 days (Figure 8B). These results indicate that heating the recombinant yeast cells makes TMOF more available to the larvae perhaps by weakening the yeast cell wall and indicate that the recombinant cells are effective in larger volumes of water (100 ml) as well as in small volume of water (1 ml). Controls that were fed non recombinant *P. pastoris* or Brewers yeast did not die (results not shown).

#### High Volume Fermentations (Industrial)

**FIGURE 9:**
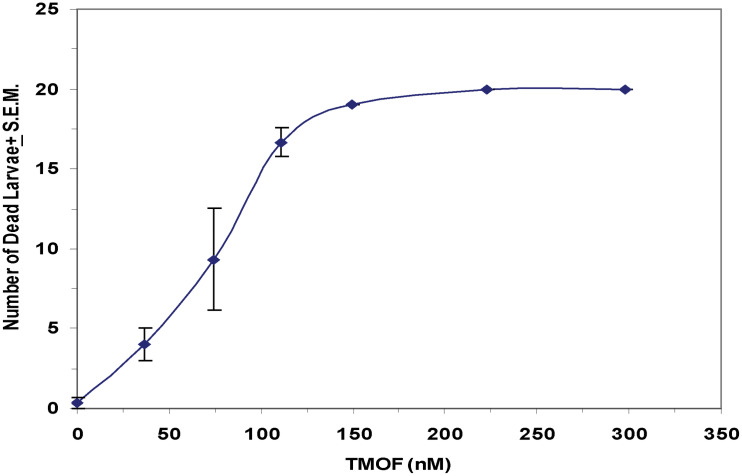
Feeding KM71H-*tmf*A cells with increasing concentrations of TMOF (0–229 nM) to *Ae. aegypti* first instar larvae after high volume (150 L) fermentation and heat treatment in an oven (entry at 210°C and exit at 85°C). Heat treated cells were fed for 12 days and mortality was daily determined. Groups of 5 larvae were tested in 25 ml sterile tissue culture wells. The amount of TMOF in heat treated cells was determined by ELISA. Results are expressed as means ± SEM.

**FIGURE 10:**
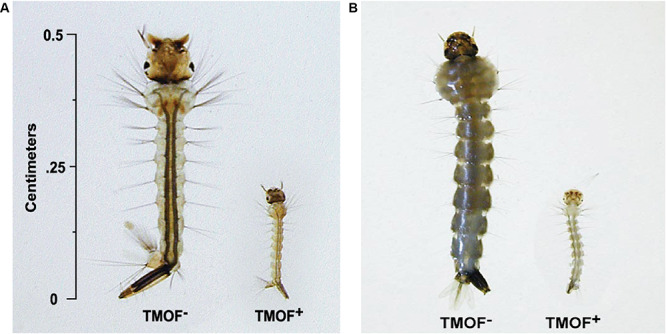
*Cx. quinquefasciatus* **(A)** and *Ae. aegypti* **(B)** larvae were fed *P. pastoris* cells KM71H TMOF^–^ (control) and KM71H-*tmf*A cells TMOF^+^ for 4 days. Larvae that fed the recombinant cells expressing TMOF did not grow because they cannot digest their food.

After high volume fermentation of *P. pastoris* KM71H-*tmf*A cell for 120 h and drying the cells in an oven (210°C entry of wet yeast cells and 85°C exit of dried cells). The cells were rehydrated in water, broken with glass beads and analyzed by ELISA for TMOF content. The recombinant cells, different number of cells (3 × 10^7^ to 1.65 × 10^8^) were fed for 10 days to 21 groups of first instar *Ae. aegypti* larvae (1 larva/ml, 20 per group) in 25 ml sterile distilled water. Mortality of the fed larvae increased as the concentration of the TMOF (nM) in the fed engineered cells increased killing all the larvae tested at concentrations larger than 100 nM, whereas cells that did not express *tmf*A (0 nM TMOF) did not kill the larvae, control (Figure 9). Similarly, feeding 16 groups (5 larvae/group) in 25 ml at TMOF concentrations of 0–229 nM (4.97 × 10^7^–1.65 × 10^8^ cells/ml) killed all the larvae at concentrations of 114 and 229 nM within 8 to 10 days, respectively (Table 3). Lower TMOF concentrations (57 nM) killed only 25% of the larvae and *P. pastoris* cells that were not engineered (control) did not kill the larvae. *Culex quinquefasciatus* larvae that were fed *P. pastoris* KM71H-*tmf*A cells for 4 days did not grow like *Ae. aegypti* larvae, and large differences of size was observed when compared with larvae that were fed control *P. pastoris* cells that were not engineered (control; Figures 10A,B). These results indicate that *P. pastoris* engineered cells expressing TMOF can be used to control larval *Ae. aegypti* and *Cx. quinquefasciatus*.

**TABLE 3:** Feeding of industrial fermented *P. pastoris* KM71H-*tmf*A to *Ae. aegypti* Larvae.

**Number of cells/ml**	**TMOF (nM)**	**Feeding days**	**Mortality (%) ± SEM**
1.65 × 10^8^	229	10	100
8.27 × 10^7^	114	8	100
4.97 × 10^7^	57	10	25 ± 3
1.6 × 10^8^ (control)	0	10	0

**Ae. aegypti* larvae 24 h after emergence, 16 groups (5 larvae per group) were fed in tissue culture wells containing 25 ml of sterile distilled water different amounts of recombinant *P. pastoris* KM71H-*tm*fA cells for 10 days. The yeast cells were fermented in 150 L fermenter for 120 h and were heat treated in oven. Larval mortality was daily followed up to 10 days. *P. pastoris* cells that were not genetically engineered were used as control.*

## Discussion

In this report, we used *P. pastoris* cells to express TMOF a decapeptide hormone that was shown to be effective against mosquito larvae when expressed in *Chlorella desiccate*, and *S. cerevisiae* (Borovsky et al., 2016, 2018). The advantage of using a methylotrophic yeast rather than *C. desiccate* and *S. cerevisiae* is the presence of a strong alcohol oxidase promoter (P*_*aox*_*_1_) and the ease in scaling up the production of heterologous genes by the cells in large fermenters. *P. pastoris* cells have shown to express high variety of different genes, from prokaryotes to higher eukaryotes (Cregg et al., 1987; Clare et al., 1991a; Brankamp et al., 1995; Fairlie et al., 2000; Fu et al., 2001). Since not all the heterologous genes are expressed at equally high level by the P*_*aox*_*_1_ due e.g., for rapid degradation of the foreign genes’ transcripts, early transcriptional terminator sequences or proteolytic degradation of the expressed protein. The biological activity of foreign proteins expressed by the cell depends on the cell wall thickness that prevents the protein from easy release from the cell, aggregation and precipitation of the foreign protein in the cell (Cassland and Jonsson, 1999). To increase the amount of heterologous protein production in *P. pastoris*, genes are inserted at the *AOX*1 locus by homologous recombination resulting in spontaneous multiple genes insertion at a low but detectable frequency (1–10%; Clare et al., 1991a, b). We transformed several *P. pastoris* cells (KM71 and KM71H) by homologous recombination using pPICZB (Figure 1A) carrying *tmf*A and *gfp-tmf*A. The *gfp-tmf*A carries an IEGR cleavage site (Figure 1B) allowing mosquito larvae to digest and release TMOF from the recombinant protein (GFP-TMOF) when it is fed to mosquito larvae, a similar strategy was also used with engineered *S. cerevisiae* cells (Borovsky et al., 2018). The engineered *P. pastoris* cells were grown on Zeocin (100–2000 μg/ml) to select colonies with up to ten insertions of *tmf*A (Figure 2B). Southern blot analyses of *P. pastoris* KM71-*gfp-tmf*A and KM71-*tmf*A and KM71H-*tmf*A confirmed that KM71 cells have a single insertion of *gfp-tmf*A or *gfp* (Figure 4), whereas KM71H-*tmf*A cells exhibited a single and 10 insertions (clones #5 and #22; Figure 5). These results confirm the genetic map of *P. pastoris* (Figure 3; Ellis et al., 1985). The Northern blot analysis of *P. pastoris* KMH71-*tmf*A cells harboring low and high copy number inserts (clones #5 and #22) show that a *tmf*A transcript (450 nt) was expressed only after the methanolic stimulation (Figure 6A). In low and high copy number cells the transcript reached a peak at 24 h, and then rapidly declined. To quantitate the abundance of the *tmf*A transcript expressed by the genetically engineered cells with a single (L) and ten insertions (H) after the Northern blot analysis (Figure 6A) the transcript ratios of *tmf*A transcript to the *act* transcript that was used as a reference gene for the Northern blot at 24 h was 7-fold higher than found in cells that were engineered with one copy of *tmf*A (Figure 6B) as was predicted by the Southern blot analysis (Figure 5). Even though the transcript levels of both low and high copy number cells declined during the fermentation the transcript ratios of cells with 10 copies to one copy cells stayed similar during the 120 h fermentation period (Figure 6B). The *tmf*A transcript level of high copy *tmf*A transcript even though it fell about 4-fold is still detected at 120 h. The transcript in low copy number cells, on the other hand, fell about 10-fold, and it almost completely disappeared and this was confirmed by ELISA determinations showing that the amount of TMOF produced in cells with high copy number is 3 to 8-fold higher than in cells expressing single *tmf*A copy (Table 2 and Figure 6A). Our observations confirm a report by Sreekrishna et al. (1988) showing a 200-fold increase in the synthesis of tumor necrosis factor in *P. pastoris* cells harboring 20 copies of the gene as compared with copy. To monitor *P. pastoris* during the methanolic fermentation and confirm during the lengthy fermentation period that TMOF is continuously synthesized, we cloned and expressed GFP-TMOF in *P. pastoris* KM71-*gfp-tmf*A cells using shake flask fermentation and at intervals (0–144 h) determined fluorescence using UV light. Cells that where illuminated with normal light did not fluoresce, whereas cells illuminated by UV light highly fluoresced (Figure 2C). To confirm that the cells synthesize GFP-TMOF, cells before induction (0 h), and cells at 93 h after induction were extracted and their proteins separated by SDS-PAGE (Figure 7) and compared with Native *Aequorea Victoria* GFP (*M*_*r*_ 27.6 kDa, Figure 7 lane 1). GFP (cycle 3) variant has a *M*_*r*_ of 30 kDA (Crameri et al., 1996) and TMOF has a *M*_*r*_ of 1.047 kDa, therefore the fusion protein GFP-TMOF has a *M*_*r*_ of 31 kDA. A strong protein band of 31 kDA was detected after 93 h fermentation (Figure 7 lane 3). In cells that were not stimulated the 31 kDa band is missing (Figure 7 lane 2). A second strong band running about *M*_*r*_ 27.6 kDa was also observed in the protein extract of the stimulated cells. This band is probably a proteolytic degradation of the GFP-TMOF at the IEGR site or other sites e.g., K5 and K26 on the GFP cycle 3 protein (Crameri et al., 1996) making the GFP-TMOF shorter by 15 or 36 amino acids the later will probably run at the observed *M*_*r*_ of about 28 kDa similar to the heavily stained band underneath the GFP-TMOF band (Figure 7 lane 3). However, only MS/MS analysis, that was not done, will confirm whether this band is indeed a proteolytic moiety of the GFP-TMOF.

The biological activity of the KM71-*gfp-tmf*A recombinant cells was followed by feeding cells to first instar larvae. A high mortality (88%) was observed at 3 days and all the larvae died at day 7. This was not due to the GFP because feeding engineered KM71-*gfp* cells (control) to the larvae did not cause mortality (Figure 8A) and GFP expressed in *S. cerevisiae* recombinant cells did not affect mosquito larvae that were fed on these cells (Borovsky et al., 2018).

Earlier biological testing of the recombinant cells was done in small volumes (1 ml) in 48 well plates. In future formulation for field applications it is important to test the recombinant cells biological activity in larger volumes that will be needed for field work. To find out the potency of the recombinant KM71-*tmf*A cells, 100 larvae were fed non treated and heat treated cells after shake flask fermentation in 200 ml water. Cells that were heat treated (55°C for 3 h) killed the larvae in 8 days, whereas cells that were tested without heat treatment killed all the larvae in 13 days (Figure 8B). These results indicate that heat treated recombinant cells are more potent. Large volume fermentation (150 L) of our recombinant KM71H-*tmf*A cells for 120 h and heat treatment of the wet cells after the fermentation in an oven in such a way that the cells entered the oven at 210°C and dry cells left at 85°C did not affect the potency of the recombinant TMOF (Tables 2, 3, and Figure 9). All the tested larvae died when fed the recombinant yeast cells that expressed TMOF (114 to 125 nM). Heat treatment of recombinant *S. cerevisiae* cells expressing TMOF also enhanced the larvicidal potency of these cells (Borovsky et al., 2018). Heating yeast cells for prolonged time releases carbohydrases and proteases that can partially hydrolyze the cell wall (Jones, 1991; Nguyen et al., 1998) making TMOF more accessible in the gut after ingestion of the recombinant yeast cells by mosquito larvae. Heat treatment did not affect TMOF or GFP-TMOF when they were tested by ELISA (Table 2) and the heat treated recombinant cells inhibited the growth of *Ae. aegypti* as well as *Cx. quinquefasciatus* larvae by starving the larvae (Figure 10).

The recombinant proteins, GFP-TMOF, and TMOF, that are produced inside the heat treated *P. pastoris* cells are protected by the yeast’s cell wall from sunlight and bacterial degradation as was reported for photosynthetic cyanobacterial species that were used for toxin delivery to control mosquito larvae (Manasherob et al., 2002; Khasdan et al., 2003). The engineered heat treated yeast cells are thus more palatable to mosquito larvae that are filter feeders and selectively eat particles found in the marsh (Clements, 1992) and could be used in the future to control mosquito larvae in the field. TMOF was found by the EPA to be safe to be used in the environment (Thompson et al., 2004; Borovsky, 2007).

## Data Availability Statement

All datasets generated for this study are included in the article/Supplementary Material.

## Author Contributions

DB and SN performed the experiments. DB and RS analyzed the data. DB designed the experiments and wrote the manuscript. All authors approved the manuscript for publication.

## Conflict of Interest

The authors declare that the research was conducted in the absence of any commercial or financial relationships that could be construed as a potential conflict of interest.
