# Supplementary figures and images for: Biochemical and Molecular Characterization of Pichia pastoris Cells Expressing Multiple TMOF Genes (tmfA) for Mosquito Larval Control
Source: Front Physiol. 2020 May 26;11:527. doi: 10.3389/fphys.2020.00527 (PMC7265970; doi:10.3389/fphys.2020.00527)

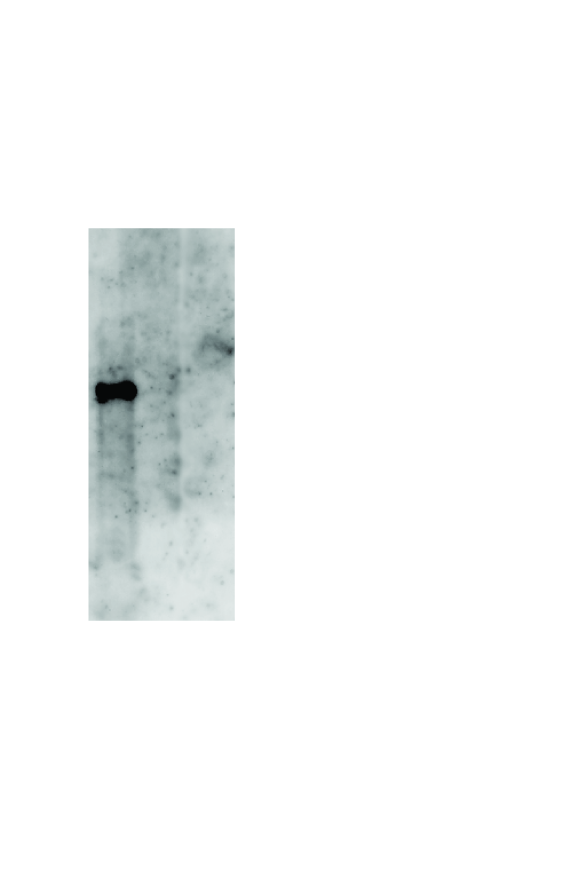

Supplement: Supplementary file 1 [file Image_1.TIF]

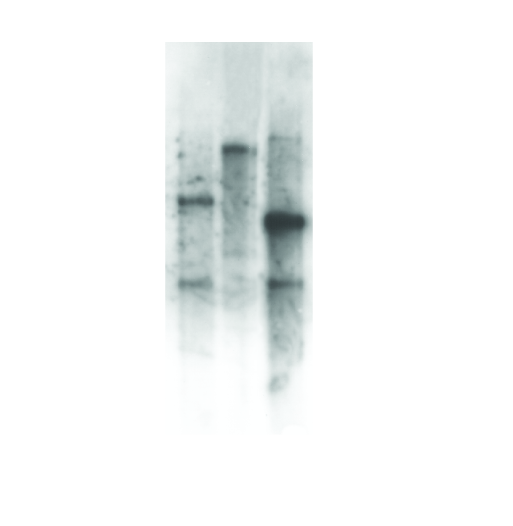

Supplement: Supplementary file 2 [file Image_2.TIF]

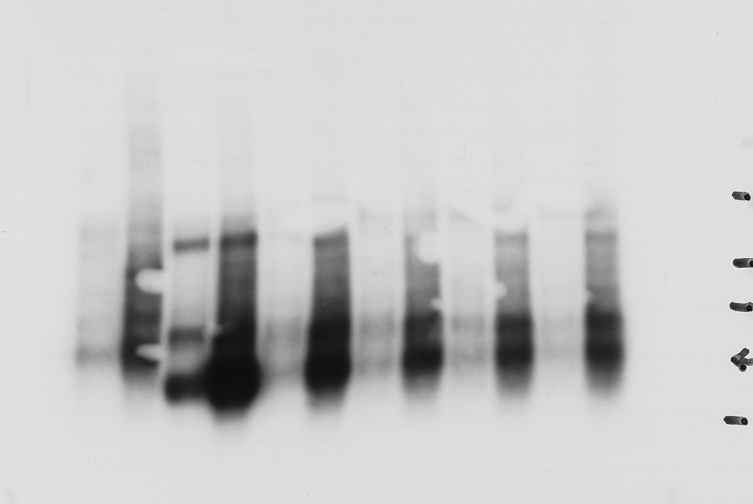

Supplement: Supplementary file 3 [file Image_3.TIF]
