# Supplementary material for: Biochemical and Molecular Characterization of Pichia pastoris Cells Expressing Multiple TMOF Genes (tmfA) for Mosquito Larval Control
Source: Front Physiol. 2020 May 26;11:527. doi: 10.3389/fphys.2020.00527 (PMC7265970; doi:10.3389/fphys.2020.00527)
Supplement: Supplementary file 4 [file Data_Sheet_1.docx]

**Supplementary material Figures captions**

**Supplementary material #1**

Original southern blot of genomic DNA of transformed KM71-*tmf*A and KM71-*gfp-tmf*A that was probed with *gfp* probe. For details see Figure 4 right panel.

**Supplementary material #2**

Southern blot of genomic DNA of transformed KM71-*tmf*A and KM71-*gfp-tmf*A that was probed with *AOX1* probe. For details see Figure 4 left panel.

**Supplementary material #3**

Original Northern blot of KM71H-*gfp-tmf*A cells (#5 and #22, low and high copy, respectively) that were fermented by shake flask for 0-120 h. For more details see Figure 6 A.
